# Risk Mapping and Situational Analysis of Cutaneous Leishmaniasis in an Endemic Area of Central Iran: A GIS-Based Survey

**DOI:** 10.1371/journal.pone.0161317

**Published:** 2016-08-30

**Authors:** Fatemeh Abedi-Astaneh, Homa Hajjaran, Mohammad Reza Yaghoobi-Ershadi, Ahmad Ali Hanafi-Bojd, Mehdi Mohebali, Mohammad Reza Shirzadi, Yavar Rassi, Amir Ahmad Akhavan, Bagher Mahmoudi

**Affiliations:** 1 Department of Communicable Disease, Deputy of Health, Qom University of Medical Sciences, Qom, Iran; 2 Department of Medical Entomology & Vector Control, School of Public Health, Tehran University of Medical Sciences, Tehran, Iran; 3 Department of Medical Parasitology & Mycology, School of Public Health, Tehran University of Medical Sciences, Tehran, Iran; 4 Zoonotic Department, Center of Disease Control (CDC), Ministry of Health and Medical Education, Tehran, Iran; University of Minnesota, UNITED STATES

## Abstract

**Introduction:**

Cutaneous leishmaniasis (CL) is among the top 10 infectious disease priorities in the world, and the leading cause of morbidity in Iran. The present study was conducted to assess the risk of CL, and to determine some epidemiological features of the disease in endemic areas of Qom Province in Central Iran during 2009 to 2013.

**Methods:**

Data regarding human cases of the disease were obtained from the Qom Province Health Center, prepared and stored in a spatial database created in ArcGIS10.3. A total of 9 out of 212 *Leishmania* spp. positive slides taken in 2013 from patients residing in Qom city were examined using molecular methods and the species of *Leishmania* was identified by PCR-RFLP. Those 9 patients had no history of travel outside the city. Spatial analysis and clustering methods were applied to find major hot spots and susceptible areas for the establishment of novel foci of the disease. Transmission patterns were examined for spatial autocorrelation using the Moran's *I* statistical application, and for the clustering of high or low values using the Getis-Ord Gi* statistics.

**Results:**

During the period of study, a total of 1767 CL cases were passively reported in the area, out of which were 65% males and 35% females. The highest and lowest numbers of cases were reported in 2010 and 2013, respectively. Importantly, 979 cases were reported from urban areas, while the remainder came from rural areas. *Leishmania major* was detected as the causative agent of CL in the city of Qom. Remarkably, most patients recorded in Qom city were associated with a history of travel to the endemic areas of CL within the province, or to other endemic areas of the disease in Iran. Spatial distribution of CL cases revealed northeastern and southwestern quarters of the city were the major hot spots of the disease (P<0.05). Hot spot and CL transmission risk analysis across the province indicated that more than 40 villages were located in high and very high risk areas of CL transmission.

**Conclusions:**

Due to the existence of hot spots (P<0.05) of CL in successive years in some quarters of Qom city, along with detection of *L*. *major* from the patients without a history of travel, there may be potential of local transmission of the disease within the city. Therefore, it is necessary to conduct a comprehensive study concerning the hot spots of CL in Qom city for curtailing the incidence of the disease in the city. The methodology and the results of this study is essential in serving as a yardstick for subsequent similar studies that will be carried out in other endemic areas of CL in Iran and providing an adequate tool for the establishment of a national database of cutaneous leishmaniasis.

## Introduction

Leishmaniasis is one of the most important vector-borne protozoal diseases and the second leading cause of morbidity, after malaria, in the world [1]. During the last decades, there has been an increasing trend of human leishmaniasis in endemic regions, and a corresponding dramatic increase in the prevalence of the disease [2]. Vector-borne diseases highly depend on environmental conditions, and changes in environmental conditions affect their transmission patterns [3]. These changes may result in the emergence of CL in non-endemic areas.

The disease exists in four different forms; among them, Cutaneous Leishmaniasis (CL) occurs in more than 90 countries worldwide, most commonly in Afghanistan, Algeria, Iran, Iraq, Saudi Arabia, Syria, Peru, Mexico and Brazil. Zoonotic Cutaneous Leishmaniasis (ZCL) in the old world occurs in semi-arid bioclimates which provide the best ecological niches for gerbils, the main reservoirs of the disease, and *Phlebotomus papatasi*, the main vector [2]. Correspondingly, urbanization and the expansion of the urban areas towards gerbil colonies, including the development of agricultural activities, have changed the pattern of the disease in recent years [3]. Despite extensive research, no vaccines have been developed for CL, and the treatment process remains problematic [4,5].

In Iran, CL exists in two forms; Anthroponotic (ACL) and Zoonotic (ZCL), and their endemic foci occur in 17 out of the 31 provinces of the country. More than 560,000 cases of the disease have been recorded during the 1983–2012 period [6,7]. Regarding the most current review, 50 species of sand flies are reported from Iran; out of which *Phlebotomus papatasi* and *Phlebotomus sergenti* s.l. act as the main vectors of ZCL and ACL, respectively [8]. Reservoir hosts of the zoonotic forms of the disease are *Rhombomys opimus*, *Meriones libycus*, *Tatera indica* and *Meriones hurrianae* gerbils in the country, whereas human and dogs serve as the reservoirs of anthroponotic form [9–11].

Spatial and spatio-temporal modeling can project a better overview of the risk maps of the disease in an area. Identification of geographical location of CL transmission is relatively challenging due to the long incubation period of the disease. Conduction comprehensive studies in endemic areas of the disease with analysis of spatial distribution of the cases in relation to environmental factors and incorporating other risk factors such as socio-economic status of the community, activity of vector(s) and/or reservoirs(s) of CL is beneficial in identifying high risk areas [12,13]. Recently, the widespread use of geographical information systems (GIS) for the analysis of risk factors associated with vector-borne diseases is commonly used in the study of leishmaniasis in Iran [12, 14–17]. To determine high-risk areas of the disease is critical in mobilizing health resources for curtailing the disease.

Qom Province is one of the important endemic foci of CL in Central Iran. Previous studies completed on the epidemiology of cutaneous leishmaniasis, its vector(s) and reservoir(s) in the province dates back to 2001. During that time, a survey in the Qanavat rural district presented a CL incidence of 2.7 and 1.4 in 2000 and 2001, respectively. *Phlebotomus papatasi* was presented as the vector of the disease, and *Meriones libycus* as the main reservoir host [18]. Following this survey, two studies were conducted; one in the suburbs of Qom city and another in the Qomrood rural district. These studies reported a scar rate of 31.4% in the area, confirming the endemicity of the disease in these areas [19,20]. Other studies have established the activity of *P*. *papatasi* in different parts of the province [21–26]. A demographic study of recorded CL cases in the province exposed an average prevalence of 25.8% [23]. Other studies identified *Leishmania major* in *P*. *papatasi*, *M*. *libycus* and human cases [22].

Uncertainly, CL transmission within the City of Qom (the capital of the Qom province) remains controversial. To resolve this inquiry, the present study was aimed at assessing the epidemiological trends of human infection, and determining the hot spots of CL incidence across Qom Province and its clusters in Qom City.

## Materials and Methods

### Study area

**Fig 1:**
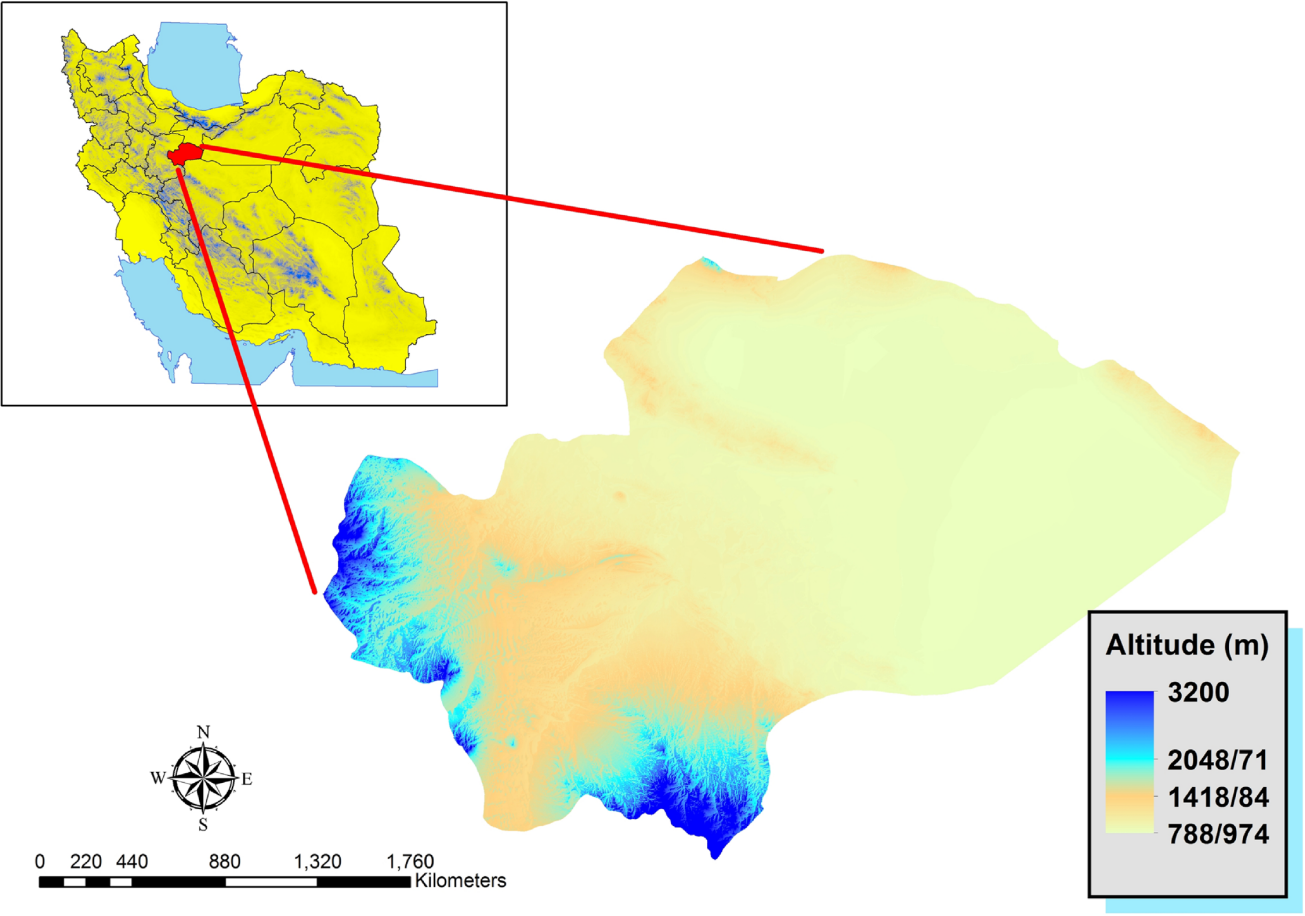
Qom Province in Iran.

Qom Province is one of the 31 provinces of Iran encompassing a land mass of 11,237 km^2^. It is located within the coordinates of 34.1828 to 34.6456° N, 50.2919 to 51.9495° E, and its provincial capital is the city of Qom (Fig 1). This province has a population of approximately 1,200,000, out of which 91.2% lives in urban areas and 8.8% in rural settings. It consists of one city, four counties, nine rural districts, and 256 villages. The climatic conditions of the province are classified into 3 types; the mountainous (western and southern parts), semi-desert (foothills and plains of central part) and hot and dry desert (Eastern parts) regions. There is a decline in the precipitation from the western to eastern borders, and across the south to the north. June and July are the warmest months within the year, with a mean maximum temperature of 38–40°C, while January is portrayed as the coldest month with a minimum average of -1 to -2°C. The eastern part of the province is covered by Salt Lake, hence, it remains a non-residential area.

### Data on human infection

Human cases of cutaneous leishmaniasis were recorded using individual patient forms which denotes essential patient’s information (age, gender, living area, number and site of lesions, date of infection, history of travel and so on) in the rural and urban health centers of Qom Province. These data are periodically reported to the provincial Health Centre and archived. We used this archived data of human infections of the disease during a five-year period of 2009–2013.

### Statistical analysis

Data regarding human infection were analyzed statistically using SPSS with Poisson regression and χ2 tests, with a 95% confidence level to find the correlation between infection of the disease and other variables such as age, gender, residential area, year and month of infection.

### Parasite detection in human lesions

Smears were taken from the patients referred to the Reference Leishmaniasis Laboratory of the Qom City Health Center in 2013. All smears were fixed, Giemsa-stained and examined carefully under light microscope with high power magnification for finding amastigote forms of *Leishmania* parasite. A total of 9 smears graded as 4+ (1–10 parasites per field) which were taken from the patients without travel history outside the city, were used for molecular detection [27]. DNA was extracted by placing the microscopic slides in separate glass jars containing ethanol (96%) for 24 hours for effective bleaching. Then, they were covered with 200 μL tissue lysis buffer (50mM Nacl, 50 mM Tris, 10mM EDTA, pH 7.4, 1% v/v Triton x-100 and 100 μg of proteinase K per ml). After a few minutes at room temperature, expansion was removed from the slides completely. The contents of the slides were then transferred into 1.5 ml reaction tubes. Cell lysis was accomplished after incubation for at least 3 h, and left overnight at 56°C [28]. The lysate was extracted by phenol-chloroform, prior to ethanol precipitation [29]. The DNA was re-suspended in 50 μl double-distilled water (DDW) and stored at 4°C [30]. Eventually, DNA was extracted using the Bioneer DNA Extraction Kit (Bioneer, Republic of Korea) under the manufacturer’s instructions, and stored at -20°C until further usage.

### ITS1 amplification and enzymatic digestion

ITS1 was amplified using specific primers; LITSR (forward: 5′-CTGGATCATTTTCCGATG-3′) and L5.8S (reverse: 5′-TGATACCACTTATCGCACTT-3′). These sets of primers amplify a fragment between 300–360 bp of *Leishmania* genome [28,29]. The post-PCR DNA products were visualized by a UV transluminator, following effective staining with ethidium bromide (safe stain) in 1.2% agarose gel electrophoresis. PCR-Ready premix (Roche, Germany) in 25 μl total reactions comprising 10 μl premix, 2 μl forward and reverse primers (10 pmol), 1 μl DNA template and 13 μl double distilled water was utilized for amplification. Iranian reference strains of *L*. *major* (Acc. No: JN860745) and *L*. *tropica* (Acc. No: EF653267) were used as positive standard controls in monitoring the reactions. RFLP analysis was deployed in identifying *Leishmania* species. Purposefully, 10 μl of the PCR product were added to 2 μl of the enzyme buffer, 1 μl of the Fast Digest Hae*III* (BsuRI) Enzyme (Fermentas, Life Sciences, Germany) and 17 μl double distilled water. The mixture was incubated at 37°C for 5–10 minutes, as recommended by the manufacturer’s protocol. Separation of the digestion products was discerned by the use of 3% agarose gels, and visualized after effective ethidium bromide staining.

### Ethical approval

This study obtained ethic permission from the Ethical Committee, Research Deputy, Tehran University of Medical Sciences (Approval Number: 911126012). All participants were provided with written informed consent forms for taking part in this study by the authors.

### Database creation

A data bank comprising the spatial distribution of human cases of CL was created and stored in ArcGIS10.3. Data of human cases of the disease were gathered from the recorded cases in the Province Health Center during 2009–2013. These data were used in mapping the distributions and spatial analyses of the disease.

### Spatial analysis of the disease occurrence

We opted to produce the incidence rates of CL across Qom Province by inverse distance weighting (IDW) interpolation. This method implements the assumption that things that are close to one another are more similar than those that are farther apart. This is especially true for an infectious disease which shows endemic foci of the disease. In this context, when the source of infection is closer, there is much likelihood of illnesses. Furthermore, the IDW interpolation technique is a common method for producing surfaces using interpolation of scatter points and has been employed in the analysis of vector borne diseases. This principle was applied to determine the spatial risk of CL in the province-based got data, and to establish the incidence rates in different urban/rural residential areas.

Cluster analysis of CL recorded cases during the study period was achieved by Moran's I index statistics [31]. Raw standardization has been done by calculating the incidence per 10000. This index can predict the clustering in similar values. Ranging from -1 to +1, the Moran's I index positive value connotes a cluster of similar values around the target feature, Zero shows spatial randomness of data, and negative values mean a feature is surrounded by non-similar features. In interpreting the results of the obtained Z-score in this analysis, 95% significance level (P<0.05) was considered.

Hot spot analysis was piloted using Getis-Ord Gi*. The G statistics have been developed to measure the distribution values [31,32]. Getis-Ord Gi* statistic provides a Z score value which resolves whether features with either high or low values cluster spatially. The larger the Z score value, the more extreme the clustering of hot spot, while a smaller Z score value predicts the clustering of low values or cold spot will to be more intense (http://resources.arcgis.com). We applied hot spot analysis on CL cases of Qom city.

## Results

### Human infection

**Fig 2:**
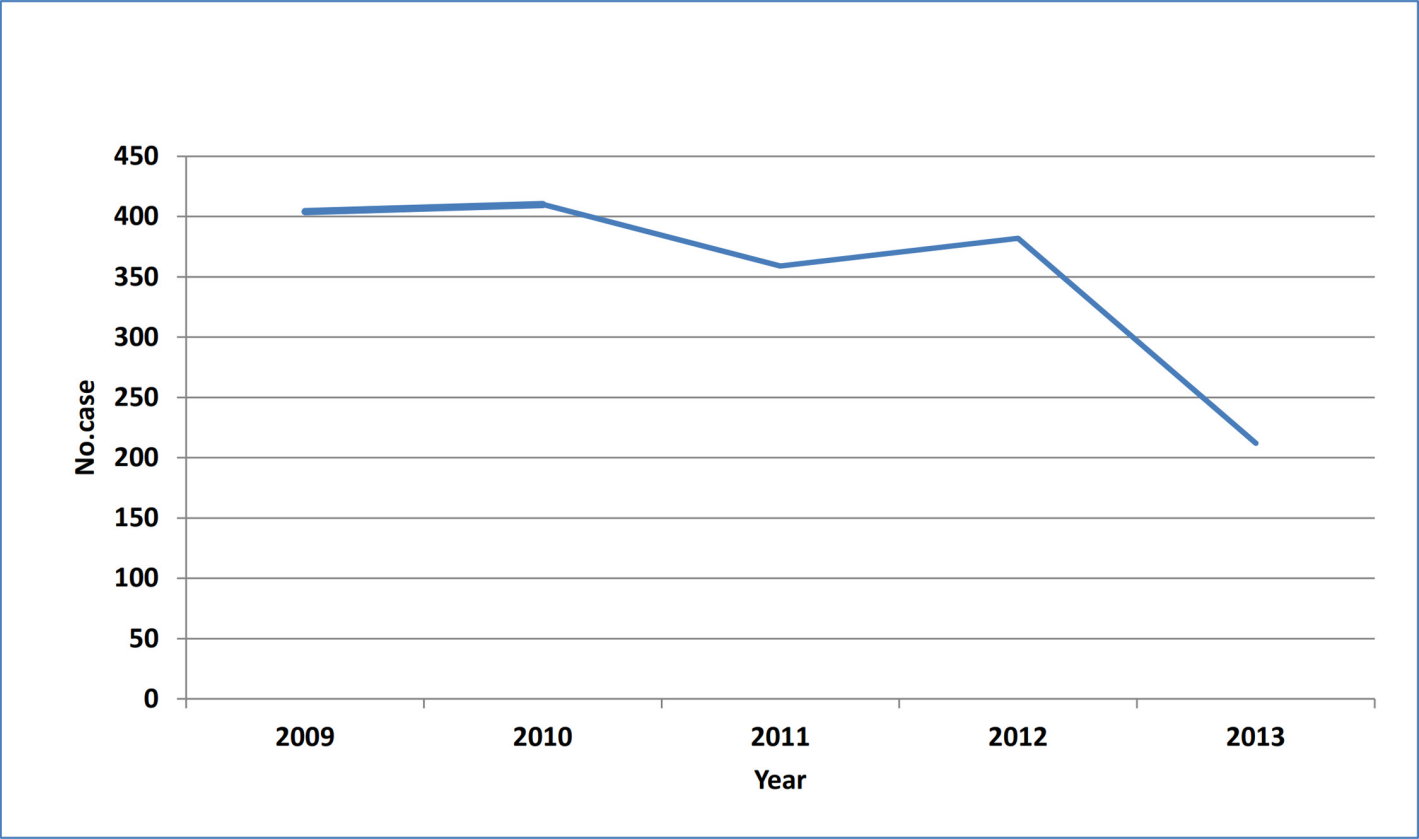
Cutaneous leishmaniasis morbidity in different years, Qom Province of Iran.

**Fig 3:**
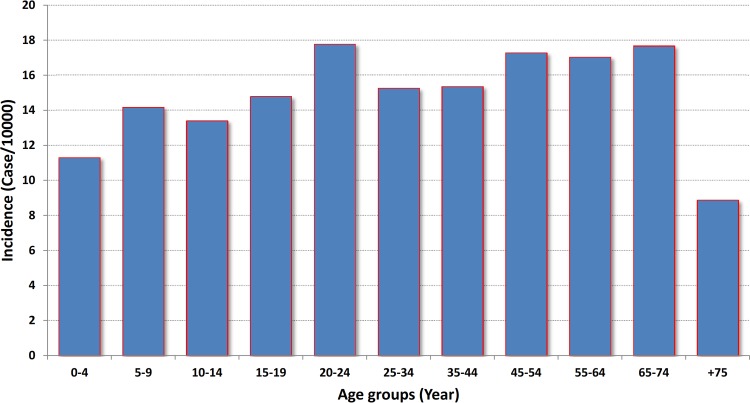
Incidence of cutaneous leishmaniasis in different age groups, Qom Province of Iran, 2009–2013.

**Fig 4:**
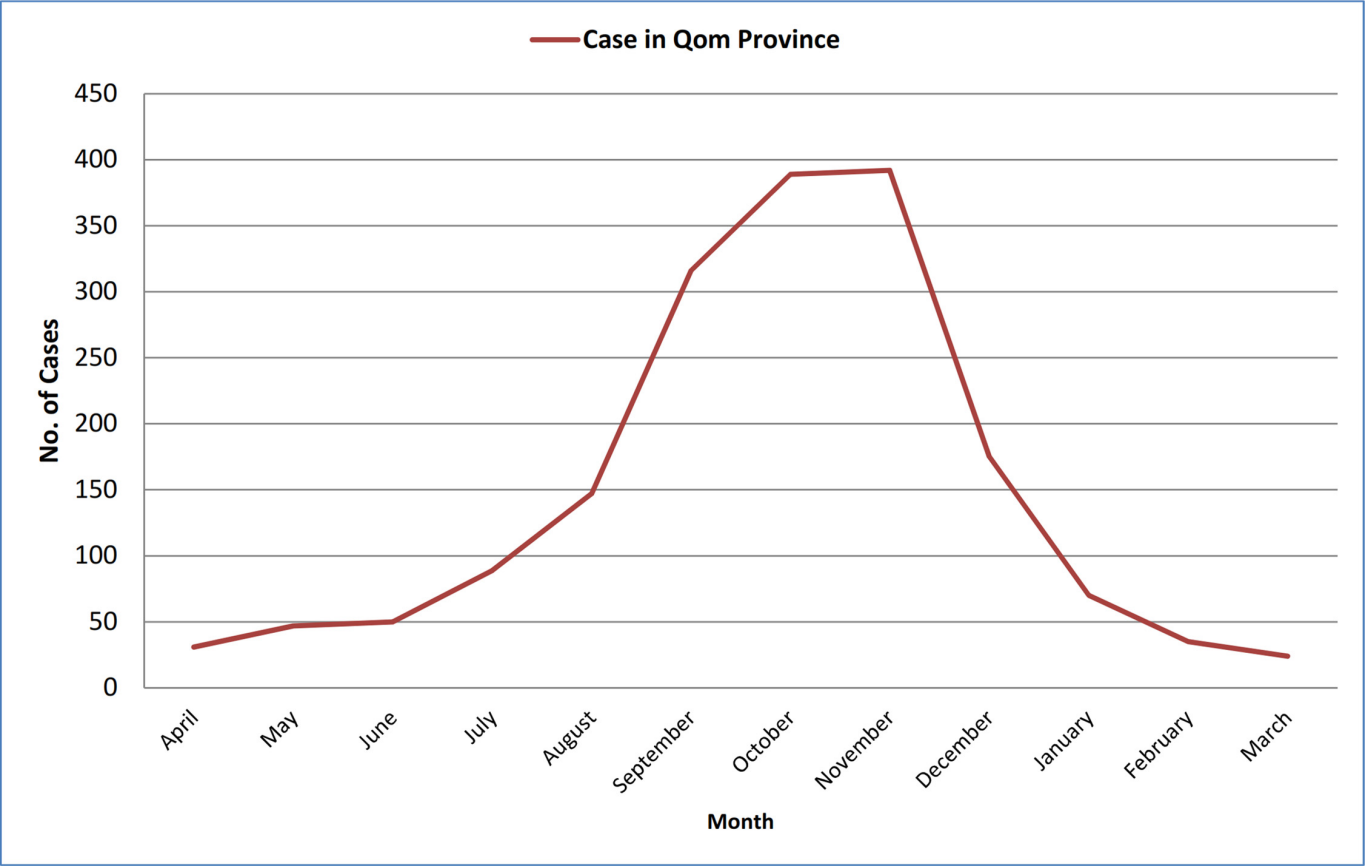
Temporal distribution of cutaneous leishmaniasis in different months of the year, Qom Province of Iran, 2009–2013.

During the five-year study period, 1767 CL cases were recorded in Qom Province. The highest and the lowest morbidity occurred in 2010 and 2013, respectively (Fig 2). There was no significant difference between human cases in different years of the study period (P>0.05). The highest incidence was calculated as 17.772 per 10000 in 20–24 age group, although it was calculated more than 17 in some other age groups (Fig 3). There was a significant difference between the chance of being infected and age groups (P<0.01). The disease was more prevalent in males rather females (P<0.01), and the number of cases in urban areas surpass those in rural places. Monthly distribution of CL cases exhibited one peak in October-November, although hits were observed in all months (Fig 4). There was no significant difference between infection rate in different months (P>0.05).

### Parasitology

**Fig 5:**
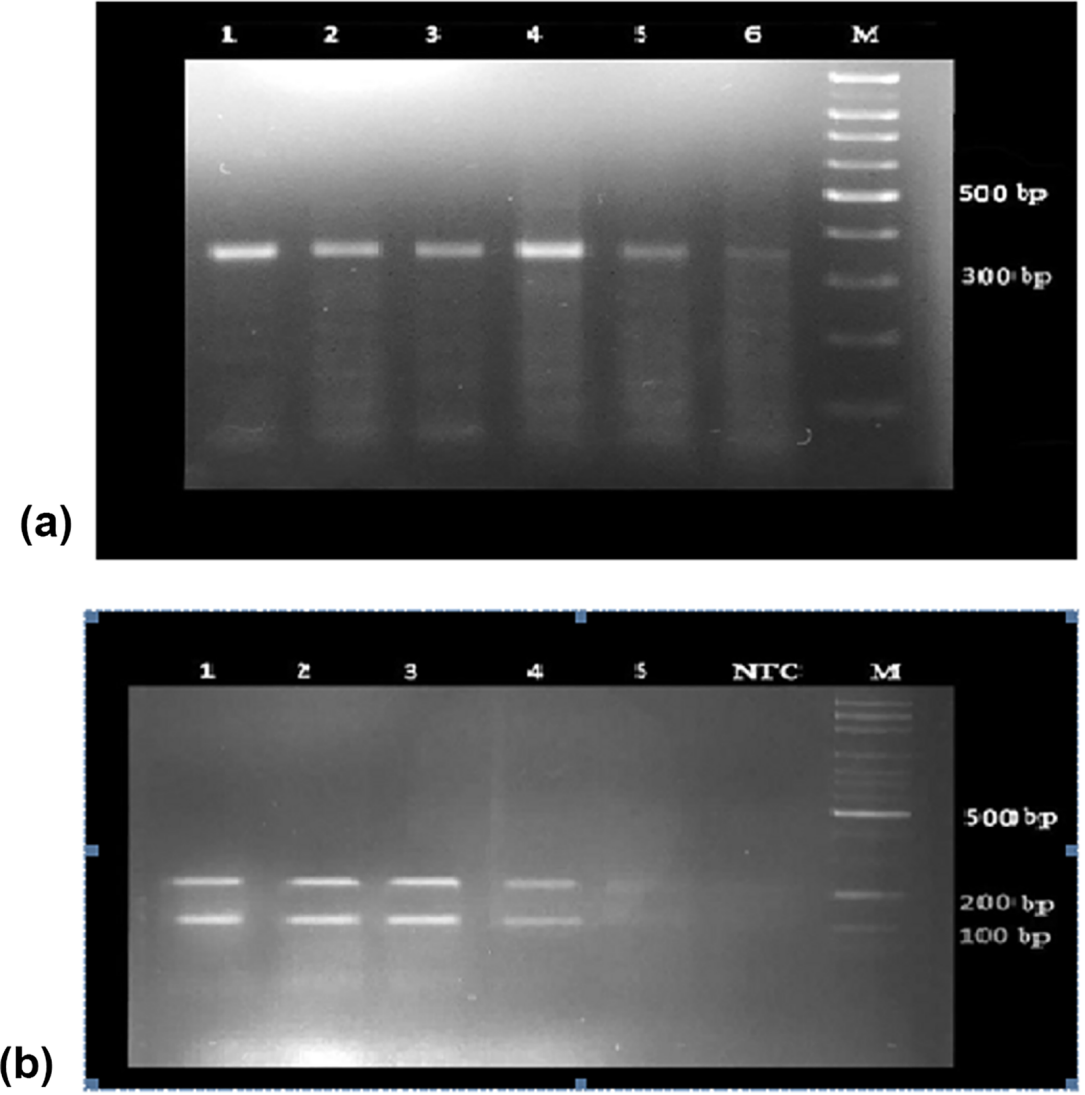
Results of PCR test for species detection of *Leishmania* parasite isolated from human lesions, Qom Province, Central Iran. (a): PCR product of *Leishmania* isolates from positive slides (Lanes 1–6); M: 100 bp size marker; (b): Electrophoresis of PCR products *Leishmania* species after endonuclease digestion with *Hae III*, Lanes 1–5: *Leishmania major*, NTC: negative test control, M: Marker (100bp).

A total of 9 slides in the grade of 4+ obtained from the patients in Qom City were used for molecular assay. Using the LITSR/L5.8S primer set for PCR, all samples were detected as *Leishmania* sp. and the same product with an expected size of 300–320 bp was observed (Fig 5A) to confirm the parasite species. The same banding pattern including the fragments of 220 and 140 bp was observed in the stained gels, after restriction enzyme digestion (Fig 5B). The results showed all samples were *Leishmania major*.

### Spatial analysis of the disease

**Fig 6:**
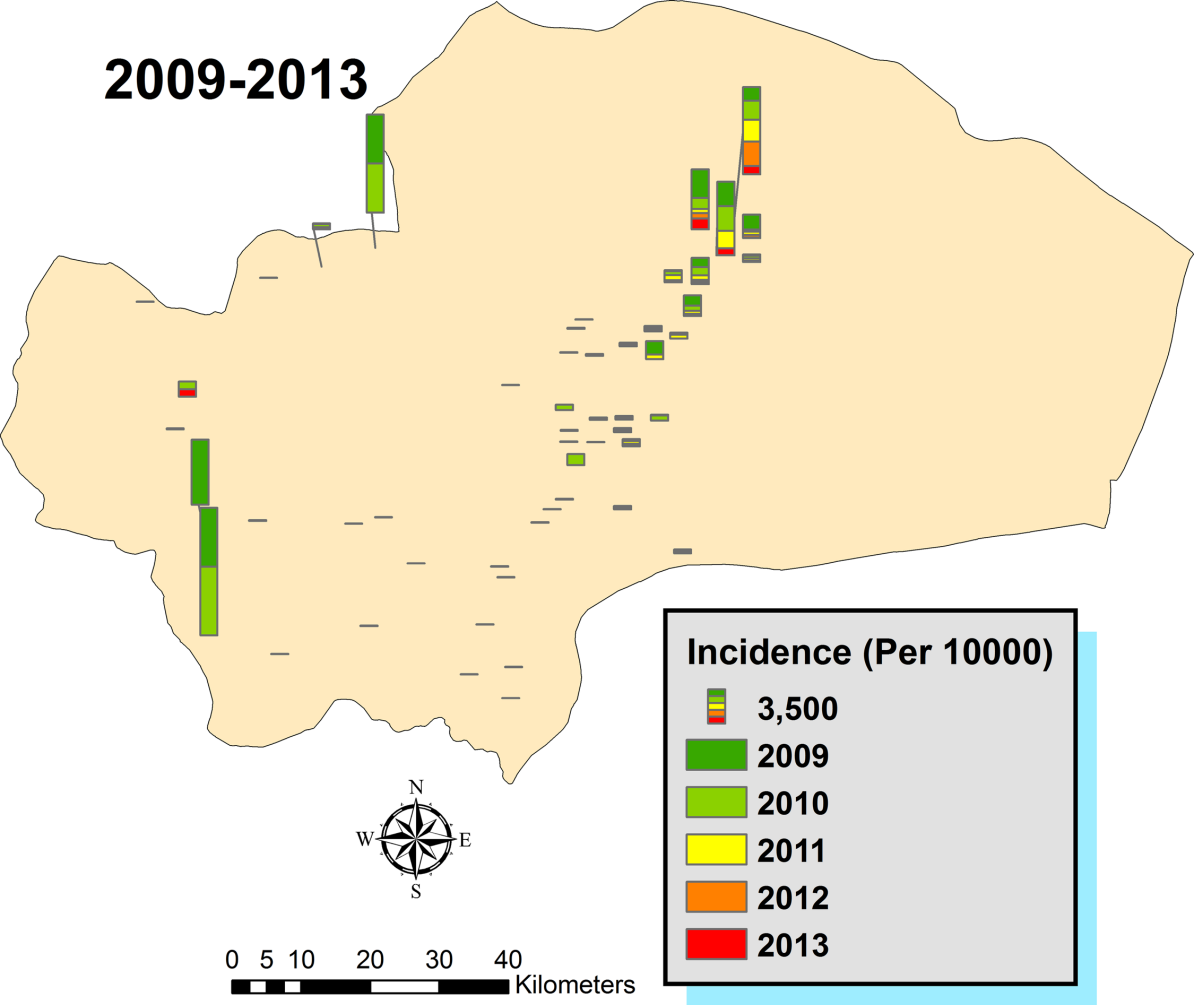
Cumulative spatial distribution of CL incidence in different residential areas of Qom Province of Iran, 2009–2013.

**Fig 7:**
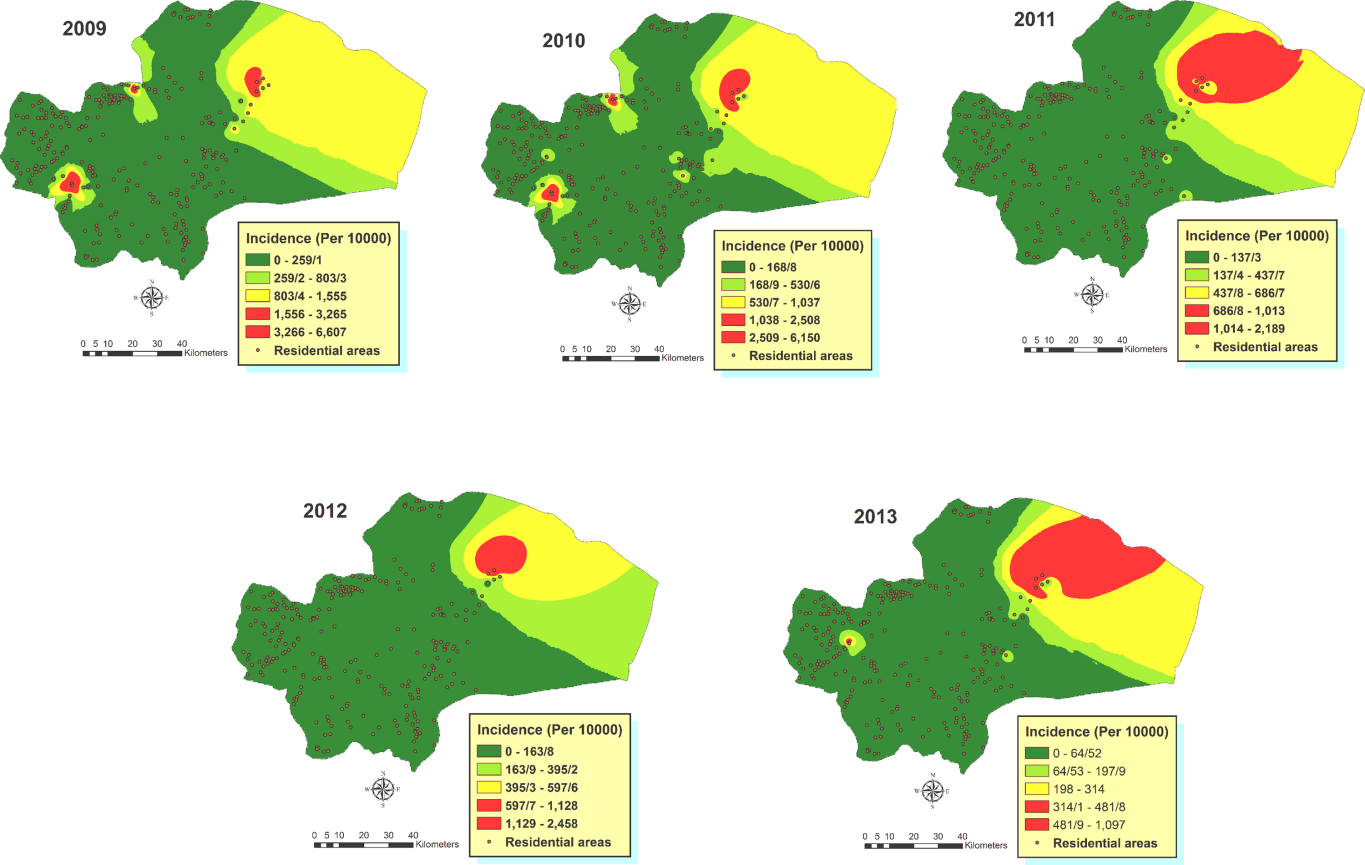
The estimated incidence of CL across Qom Province of Iran, 2009–2013.

Spatial distribution of the disease incidence across the urban/rural residential places of the province was mapped (Fig 6). Larger columns illustrate the most infected areas are present in the central and eastern part of the province in the recent years although during 2009–2010 a high incidence was also recorded in 3 villages at the northern and western areas. Overall, the number of cases was higher in the eastern part of Qom province, in some villages where agricultural activities are well developed. Data of CL cases were transferred to the spatial databank of the province, and the areas with the greatest probability of existing CL cases were validated by the IDW analysis of the incidence in different years (Fig 7). The results affirm the highest incidence occurred in rural areas of northeastern parts of the province, although higher incidence arose in other parts of some villages, especially in 2009–2010. It seems that infected areas were expanded in 2013, considering the incidence of the disease.

**Fig 8:**
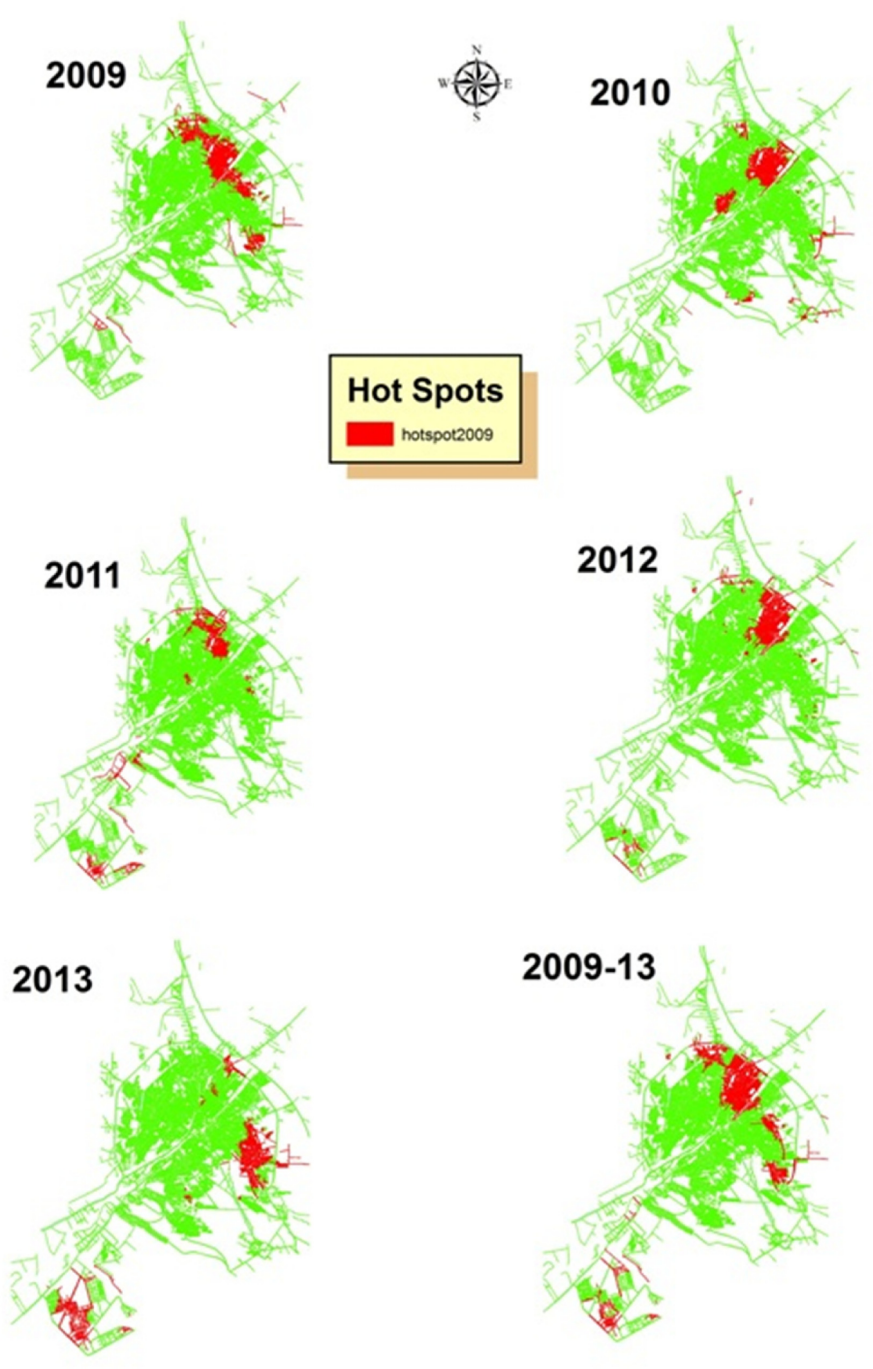
Hot spot clusters of CL in Qom City, Central Iran, 2009–2013.

The results of clustering hot spots in Qom city display hot spots of CL are located in northeastern and southwestern quarters. Red clusters show Gi Z Score > 1.96 (P<0.05), (Fig 8). Northern and Eastern quarters are close to the agricultural fields, but southwestern quarter (Pardisan) harbors a newly established huge housing project.

The z-scores computed for Moran's I index in different years of the study period in Qom city was realized to be 2.170575–3.733071, with p-values <0.05 (Table 1). This confirms the clustering of the disease pattern in the city, with less than 5% likelihood that this clustered pattern could occur by chance. Considerably, the high/low clustering (Getis-Ord General G) resulted in a Z-score range of 2.014262–3.459868 for different years of the study period. Conforming to the null hypothesis for the High/Low Clustering (General G) statistic, which states that there is no spatial clustering of feature values, high-clustered pattern was maintained in all years (P<0.05).

**Table 1:** Spatial autocorrelation estimation of different years using Moran's I index and General G statistics in Qom City, Central Iran, 2009–2013.

Year	Moran's I	Z-score	General G	Z-score	Pattern
**2009**	0.003109	2.972219	0.000089	2.758019	High-Clustered
**2010**	0.002609	2.512780	0.00142	2.137591	High-Clustered
**2011**	0.002258	2.170575	0.00085	2.014262	High-Clustered
**2012**	0.003478	3.317438	0.000088	3.108197	High-Clustered
**2013**	0.003918	3.733071	0.000107	3.459868	High-Clustered

Significant (P<0.05)

## Discussion

Qom province is one of the endemic foci of CL in Iran. Our findings support this claim, such that cases of cutaneous leishmaniasis were recorded in all age groups, while the incidence of the diseases was different significantly (P<0.01). Despite the disease portrayed as a public health problem with several epidemics in some parts of the province, this is the first applied research in using geographical information system for establishing database of cutaneous leishmaniasis in Qom Province, and one of the novel research ever conducted in this discipline in Iran.

Previous studies examining the agent of CL in Qom Province presented *L*. *major* isolation in rural areas [18,22]. Our findings are consistent with those of previous studies. *Leishmania major* is the causative agent of CL in different foci of ZCL in Iran [11]. Our results were also in agreement with these studies. A literature review shows Qom City was considered as one of the ACL foci of Iran in the past. Although most CL cases in the city were associated with a history of travel in or out of the province to endemic foci of the disease, however, because there are hot spot clusters of CL in some quarters, supported by the detection of *L*. *major* in lesions of patients, the determination of activity of *M*. *libycus* and *Nesokia indica* in agricultural fields within the suburbs of the city [20], and the identification of both *P*. *papatasi* and *P*. *sergenti* s.l., as vectors of ZCL and ACL, respectively [26] from different parts of the city, it can be deduced that the epidemiology of the disease has changed. Besides the travelling of more than 12 million pilgrims (at-risk population) to Qom City, citizens of this area have also been traveling to different parts of Iran, especially to ZCL focus of Natanz and ACL focus of Mashhad. Therefore, ACL can be transmitted within the city from imported cases. Hence, it is crucial to combat the current situation and obstruct the transmission cycle of CL in the city.

Previous studies performed in this province have confirmed the activity of 20 species of sand flies, and *L*. *major* has been detected as the causative agent, with *P*. *papatasi* serving as the main vector of ZCL in the province [18,26]. This sand fly is well-known as the main vector in different foci of ZCL in Iran [6,8,11,16]. Studies on the fauna of rodents in different sentinel sites of Qom Province showed *M*. *libycus* as the dominant species in the endemic foci of CL, and *L*. *major* has been isolated from this gerbil [22]. *Meriones libycus* was encountered as the main reservoir host of cutaneous leishmaniasis in endemic foci of the disease in our study area [10] and some other foci of the disease in the country [11]. Occurrence of both the vector and the reservoir near human residential places should be considered to prevent the disease transmission. Concurrently, *L*. *major* was detected from human lesions in our study. This parasite circulates in the above-mentioned vector and reservoir.

The forefront of this study was to establish a spatio-temporal database for cutaneous leishmaniasis in the study area as a foundation for research and evidence-based decision-making system. This data base may be updated annually with new records of the disease and improved with other relevant data of CL including those of the vectors and reservoir hosts, which can be used for mapping and highlighting disease-prone areas and the population at risk. This will make sure a more effective and prompt preventive and control strategies. Likewise, future modeling and prediction of CL will be much easier using environmental and climatic data.

The results obtained from smoothed CL incidence rates and inverse distance weighting (IDW) analysis showed low and high endemic areas across the province during the study period. This method has been previously used to predict spatial incidence of VL in India [33], although some other studies have used different approaches for modeling risk of leishmaniasis [34,35]. It can be observed that the high endemic areas are in northeastern part of the province in all years although there are a number of villages in the western and northern regions of the study area with high incidence of the disease during 2009–2010. After 2 years, western parts of the province were characterized by an increase in the incidence rate, and should be considered as an unstable area for CL transmission.

Hot spots of CL were discovered in some quarters of the city. This information assists in the prediction of transmission of the disease within the city. Therefore to prevent epidemics, especially in newly established quarter of Pardisan which has huge housing projects, effective preventive programs are recommended. Our findings suggest a comprehensive study should be done on the current epidemiology of CL in hot spots of Qom city; to use this study as a template for CL studies in the country; to hold regular training programs about CL in improving the knowledge of people living in at-risk areas of the province; to establish a comprehensive online national databank for recording CL cases in the country; and to stratify the risk of cutaneous leishmaniasis, not only based on the morbidity, but also the possibility of the distribution the of vectors and reservoirs in Iran.

## Conclusions

This study confirms local transmission of CL within the Qom City in two different situations: first, an old quarter next to the agricultural fields, where both vector and reservoir of ZCL has been reported; and second, a newly established residential area with building projects under construction that had provided a suitable environment for breeding of sand flies in debris, and the potential of CL transmission to the workers and people living in these areas. High risk areas for CL transmission were identified across the Qom Province. Spatial and statistical analysis of GIS analyzed the situation of a serious health issue and identified the hot spots areas of the disease. Also, detection of *L*. *major* from the patient with no history of travel in some quarters of Qom City which confirms local transmission can be considered for planning new activities to control CL within the city, where there are uncertainties about local transmission in which all cases have been considered as imported.

## Supporting Information

S1 Data Raw data used for this study. (XLSX)

S1 File Charts used within the paper. (XLSX)
